# Fatostatin reverses progesterone resistance by inhibiting the SREBP1-NF-κB pathway in endometrial carcinoma

**DOI:** 10.1038/s41419-021-03762-0

**Published:** 2021-05-26

**Authors:** Xiaohong Ma, Tianyi Zhao, Hong Yan, Kui Guo, Zhiming Liu, Lina Wei, Wei Lu, Chunping Qiu, Jie Jiang

**Affiliations:** 1 Department of Gynecology and Obstetrics, Qilu Hospital of Shandong University, 250012 Jinan, China; 2 Gynecologic Oncology Key Laboratory of Shandong Province, Qilu Hospital of Shandong University, 250012 Jinan, China; 3 Department of Obstetrics and Gynecology, Women and Children’s Hospital, Decheng district Dezhou, Shandong, 253017 P.R. China

**Keywords:** Transcriptional regulatory elements, Cancer therapeutic resistance

## Abstract

Progesterone resistance can significantly restrict the efficacy of conservative treatment for patients with endometrial cancer who wish to preserve their fertility or those who suffer from advanced and recurrent cancer. SREBP1 is known to be involved in the occurrence and progression of endometrial cancer, although the precise mechanism involved remains unclear. In the present study, we carried out microarray analysis in progesterone-sensitive and progesterone-resistant cell lines and demonstrated that SREBP1 is related to progesterone resistance. Furthermore, we verified that SREBP1 is over-expressed in both drug-resistant tissues and cells. Functional studies further demonstrated that the inhibition of SREBP1 restored the sensitivity of endometrial cancer to progesterone both in vitro and in vivo, and that the over-expression of SREBP1 promoted resistance to progesterone. With regards to the mechanism involved, we found that SREBP1 promoted the proliferation of endometrial cancer cells and inhibited their apoptosis by activating the NF-κB pathway. To solve the problem of clinical application, we found that Fatostatin, an inhibitor of SREBP1, could increase the sensitivity of endometrial cancer to progesterone and reverse progesterone resistance by inhibiting SREBP1 both in vitro and in vivo. Our results highlight the important role of SREBP1 in progesterone resistance and suggest that the use of Fatostatin to target SREBP1 may represent a new method to solve progesterone resistance in patients with endometrial cancer.

## Introduction

Endometrial carcinoma (EC) is the most common form of malignant gynecological tumor, ranking in fourth and sixth place of all cancers with respect to morbidity and mortality, respectively^1^. There is an apparent trend for younger patients to be affected by EC; these patients are normally inclined to select conservative forms of treatment so as to maintain their fertility. Conservative progesterone-based treatment is also an effective treatment for advanced and recurrent endometrial cancer. A phase II clinical trial revealed that ~55% of EC patients showed a complete response (CR) to conservative forms of treatment that included medroxy-progesterone acetate (MPA), with a 47% recurrence rate between 7 and 36 months^2,3^. Nevertheless, other studies have shown that in excess of 30% of EC patients do not respond to MPA and acquire resistance to progesterone during the course of progesterone treatment^4,5^. Data therefore suggest that progesterone resistance appears to restrict the efficacy of conservative treatment, although the specific molecular mechanism involved still needs to be identified.

Sterol regulatory element-binding proteins (SREBPs) are transcription factors that regulate the anabolism of cholesterol and lipids. SREBPs activate the transcription of target genes by binding with the sterol regulatory elements of the lipid synthase gene promoter or enhancer^6,7^. SREBPs can be further subdivided into three isoforms: SREBP-1a, SREBP-1c, and SREBP-2. SREBP1 has been found to be abnormally expressed in a variety of human tumors, including prostate carcinoma, breast carcinoma, and hepatocellular carcinoma^8–10^. In addition, it has also been reported that a reduction in the levels of sterols in tumor cells will cause SREBP1 to be synthesized into precursors within the endoplasmic reticulum (ER), thus creating protein complexes with SREBP-cleavage-activating protein (SCAP). These complexes are then transported to the Golgi apparatus for hydrolysis, thus releasing SREBP1 into the nucleus and activating the transcription of enzymes related to cholesterol and fatty acid anabolism^11–13^. When the levels of sterols increase, the progesterone receptor (PGR) membrane component/insulin inducible gene (PGRMC/INSIG) complex will block the transportation of SREBPs-SCAP from the ER to the Golgi apparatus by binding with SCAP; this reduces the production of lipids and cholesterol^14,15^. Our research group has been interested in SREBP1 for a substantial period of time and has verified that SREBP1 promotes the occurrence and development of EC^16,17^. According to previous research, SREBP1 has the proven ability to participate in colorectal cancer and renal clear cell carcinoma by regulating the NF-κB pathway^18,19^. These findings motivated us to investigate whether SREBP1 regulates the NF-κB pathway in cases of progestin resistance.

Fatostatin (formerly referred to as 125B11) is a diarylthiazole derivative that is capable of blocking the ER-to-Golgi transport of SCAP, thereby inhibiting the activation of SREBP^20,21^. Previous mechanistic research has demonstrated that Fatostatin combines directly with SCAP, thus blocking Golgi-specific glycosylation on SCA. This prevents SCAP from exiting the ER, thus inhibiting the growth of cells that lack SCAP^22^. Other research studies have shown that Fatostatin exerts anti-cancer effects in prostate, breast, and EC, by inhibiting the activation of SREBP1^23–25^. Therefore, we hypothesized that Fatostatin can reverse progesterone resistance in EC by inhibiting SREBP1.

In the present study, we investigated the mechanisms associated with the action of Fatostatin on progesterone resistance by carrying out a range of experiments in vivo and in vitro. Our eventual goal is to develop a new therapeutic strategy for the clinical treatment of EC.

## Results

### SREBP1 expression was increased in progesterone-resistant endometrial samples after progesterone treatment

**Fig. 1:**
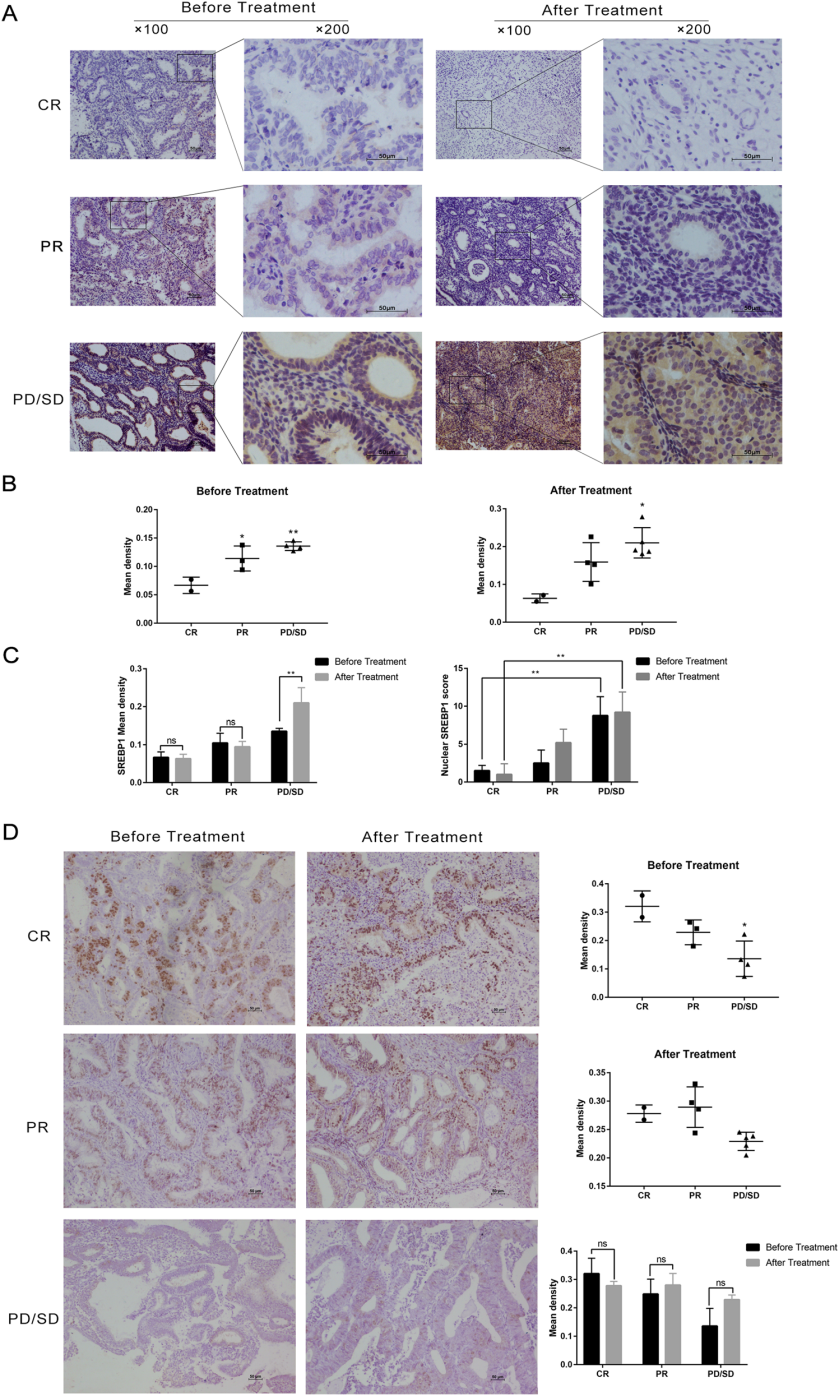

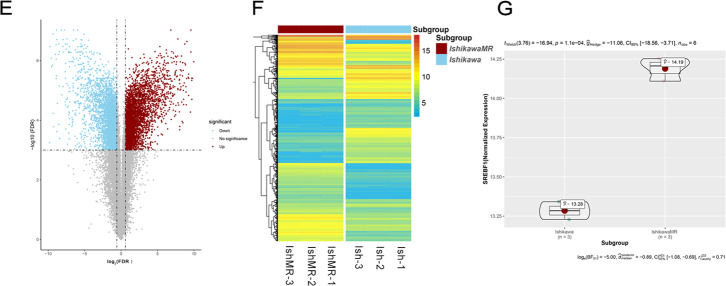
Expression of SREBP1 and PGR in progesterone resistance samples and microarray verification in cell lines. **A** Representative images of IHC staining of SREBP1 in endometrium before and after progesterone treatment in CR, PR and PD/SD group. **B** SREBP1 IHC scores in endometrium before and after progesterone treatment were analyzed by Image-Pro Plus 6.0. **C** The SREBP1 intensities of IHC staining were quantitated by Image-Pro Plus 6.0. The intensity of nuclear SREBP1 was graded as follows: 1, weak; 2, moderate; and 3, strong. The Score = (percentage of cells of weak intensity × 1) + (percentage of cells of moderate intensity × 2) + (percentage of cells of strong intensity × 3). **D** Representative images of IHC staining of PGR expression in endometrium before and after progesterone treatment in CR, PR, and PD/SD group. The intensities of IHC staining were quantitated by Image-Pro Plus 6.0. **E** Volcano plot of RNA-seq from microarray data. The red dots represent upregulated DEGs based on a fold change of >1.5. The volcano plot displays differential genes when comparing IshMR cell lines and Ish cell lines. **F** Hierarchically clustered heatmap of differentially expressed genes in IshMR and Ish cells. **G** The violin plot depicting the expression distribution of SREBP1 between IshMR and Ish cells. CR complete response, PR partial response, PD/SD progressive/stable disease, Ish Ishikawa, IshMR MPA-resistant cell line of Ish cells. *P < 0.05, **P < 0.01, ***P < 0.001, and ****P < 0.0001 for statistical analysis of the indicated groups.

Analyses showed that the expression of SREBP1 was directly related to progesterone resistance. Progesterone treatment resulted in a CR in three patients, a partial response (PR) in six patients, and progressive/stable disease (PD/SD) in seven patients. The staining of clinical specimens in the three groups before and after treatment showed that SREBP1 was expressed at low levels in the CR group. In contrast, the levels of SREBP1 were increased in the PR group, although there was no significant difference when comparing before and after treatment. Furthermore, the expression of SREBP1 in the PD/SD group was significantly higher after treatment (Fig. 1A–C). Nuclear staining of SREBP1 confirmed a similar pattern of results. Figure 1A shows a representative sample of each group; some areas are shown at higher levels of magnification to facilitate observation of the nuclear staining of SREBP1. The presence of the PGR is now widely believed to be a prerequisite for the progesterone response. We therefore investigated the expression of the PGR in clinical samples and found that the progesterone-resistant samples expressed lower levels of the PGR. However, there was no significant difference in terms of the PGR when compared between before and after progesterone treatment (Fig. 1D).

### SREBP1 was upregulated in progesterone-resistant cells

We carried out microarray analysis in the Ishikawa (‘Ish’) cell line and an MPA-resistant cell line of Ish cells, which we refer to here in as IshikawaMR (‘IshMR’). The raw data arising from this analysis has been uploaded to the GEO database (reference: GSE121367). The microarray data were preprocessed and normalized based on the Bioconductor platform in order to obtain a gene expression matrix. The limma R package was then used to perform differential expression analysis between the two cell lines. The threshold was set at a false discovery rate (FDR) < 0.001 and a fold change > 1.5 or <−2/3. A volcano plot was created to show the differentially expressed genes. A heat map was also created to show the distribution of differentially expressed genes between groups. A Violin plot was used to demonstrate that the expression of SREBP1 in the IshMR cell line was higher than that in the Ish cell line (*P* < 0.0001); these findings were consistent with those from clinical samples (Fig. 1E–G).

**Fig. 2:**
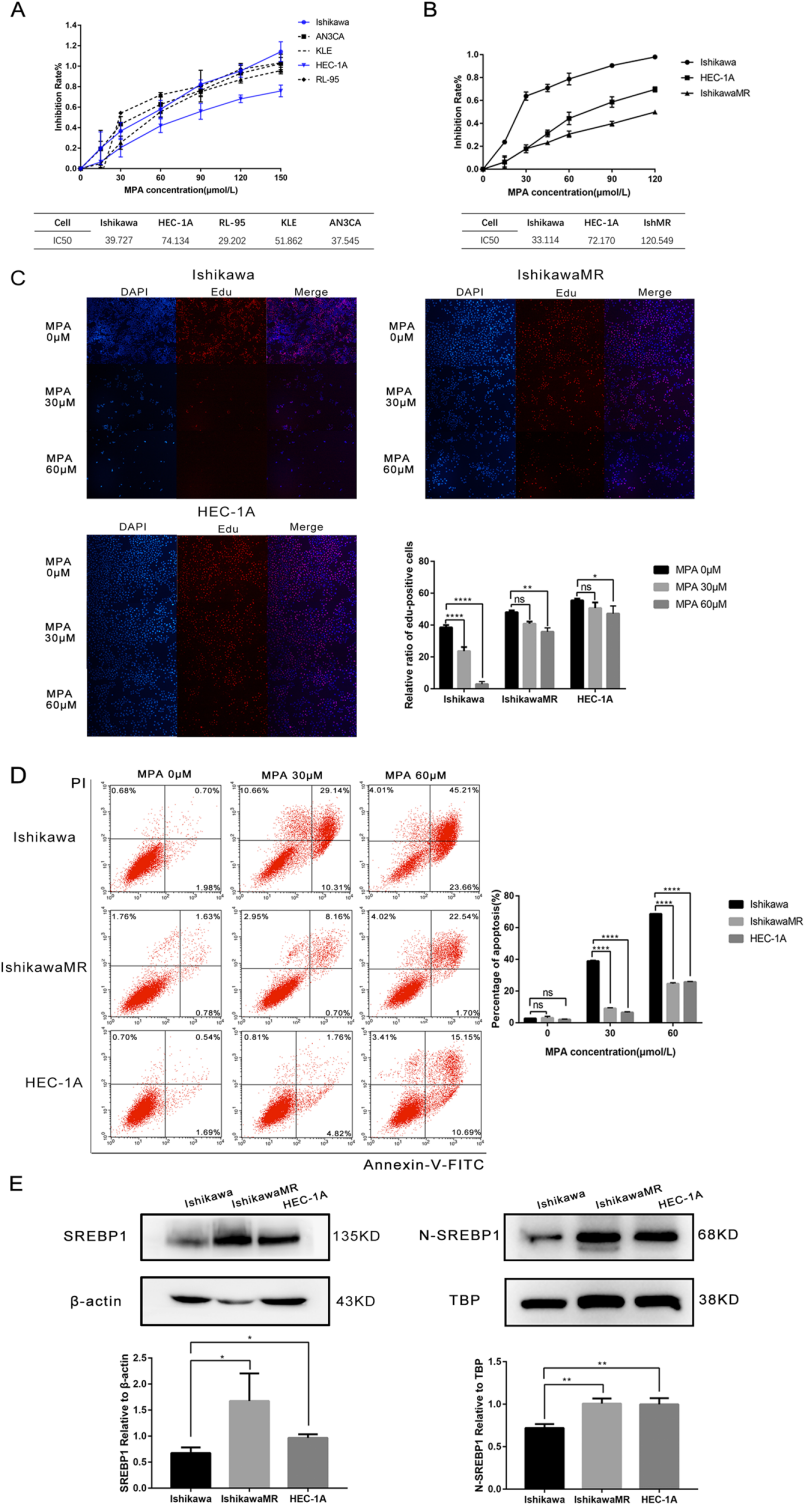

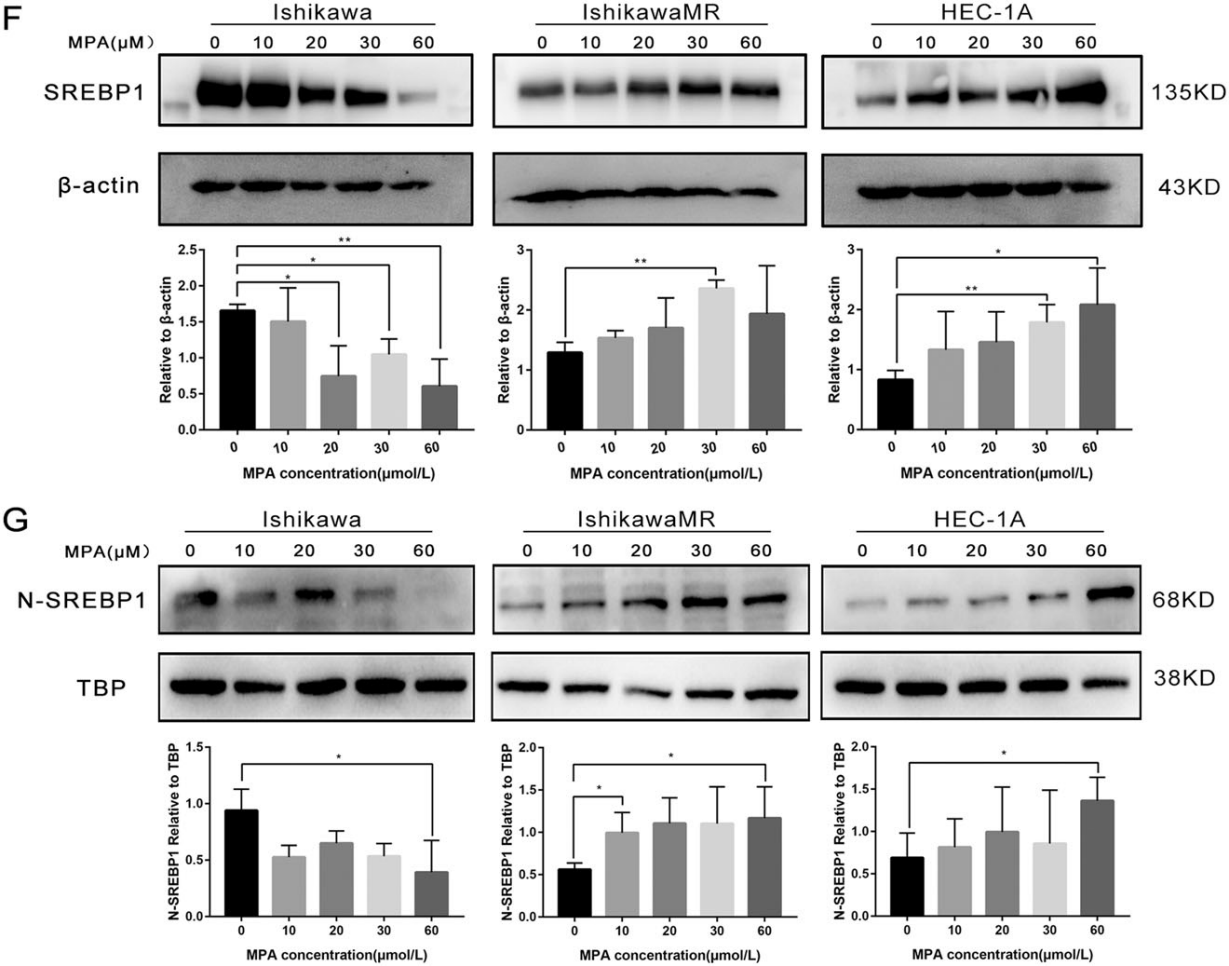
Characterization of endometrial carcinoma cells for progesterone and high expression of SREBP1 in progesterone-resistance cells. **A** Growth curves of 5 endometrial cancer cells maintained in different-dose MPA for 48 h and IC50 of 5 endometrial carcinoma cells for MPA. The growth curves were analyzed using a two-way ANOVA test. **B** Growth curves of Ish, IshMR, and HEC-1A maintained in different-dose MPA for 48 h and IC50 of 3 endometrial carcinoma cells for MPA. **C** EDU incorporation assay after incubation with 0, 30, 60 μM MPA respectively for 48 h. **D** Flow cytometry assay after incubation with 0, 30, 60 μM MPA respectively for 48 h. **E** Full-length and nuclear SREBP1 protein level in Ish, IshMR, and HEC-1A. **F** The expression of full-length SREBP1 in Ish, IshMR, and HEC-1A cells with different-dose MPA treatment. **G** The expression of nuclear SREBP1 in Ish, IshMR, and HEC-1A cells with different -dose MPA treatment. All experiments were repeated three times at least. *P < 0.05, **P < 0.01, ***P < 0.001, and ****P < 0.0001 for statistical analysis of the indicated groups. Quantitation of western blotting assay bands shown in using Image J. Statistical analysis was performed using Student’s *t* test.

Five endometrial cancer cell lines (Ish, HEC-1A, RL-95, KLE, and AN3CA) were cultured in a medium with different doses of MPA. MTT assays showed that MPA inhibited the growth of endometrial cancer cells in a dose-dependent manner. The IC_50_ values were 39.727, 74.134, 29.202, 51.862, and 37.545, respectively (Fig. 2A). The Ish, HEC-1A, and IshMR, cell lines were selected as progesterone-sensitive cells, primary progesterone-resistant cells, and secondary progesterone-resistant cells, respectively, for subsequent experiments. Data arising from the MTT assays and EDU experiments revealed that the inhibitory action of MPA on survival and proliferation was concentration dependent for Ish, HEC, and IshMR cells; the IC_50_ was 33.114, 72.170, and 120.549, respectively (Fig. 2B, C). Cell apoptosis experiments revealed a significantly higher rate of apoptosis in the Ish cells than the IshMR and HEC-1A cells as the concentration of MPA was increased (Fig. 2D). Protein expression detection in Ish, IshMR, and HEC-1A cells, indicated that the expression levels of the full-length SREBP1(135 kD) protein and the nuclear SREBP1 (68 kD) protein were all increased in the two cell types that were resistant to progesterone (Fig. 2E). Following progesterone stimulation, SREBP1 expression was detected in all three cell types. Quantitative analysis showed that the administration of MPA over 12-hours at concentrations of 10, 20, 30, and 60 μM, resulted in a reduction of SREBP1 expression in Ish cells by 15.1%, 26.7%, 31.1%, and 78.1%; however, SREBP1 expression increased by 0.8-, 0.9-, 1.3-, and 4.2-fold in the HEC-1A cells and by 0.2-, 0.5-, 0.8-, and 1.1-fold in the IshMR cells, respectively (Fig. 2F). Following nucleoprotein extraction, we found that the expression of the nuclear SREBP1 protein also showed the same trend (Fig. 2G).

In conclusion, the overexpression of SREBP1 was related to the occurrence and development of progesterone resistance in EC.

### SREBP1 promoted cell proliferation, inhibited apoptosis, and induced progesterone resistance

**Fig. 3:**
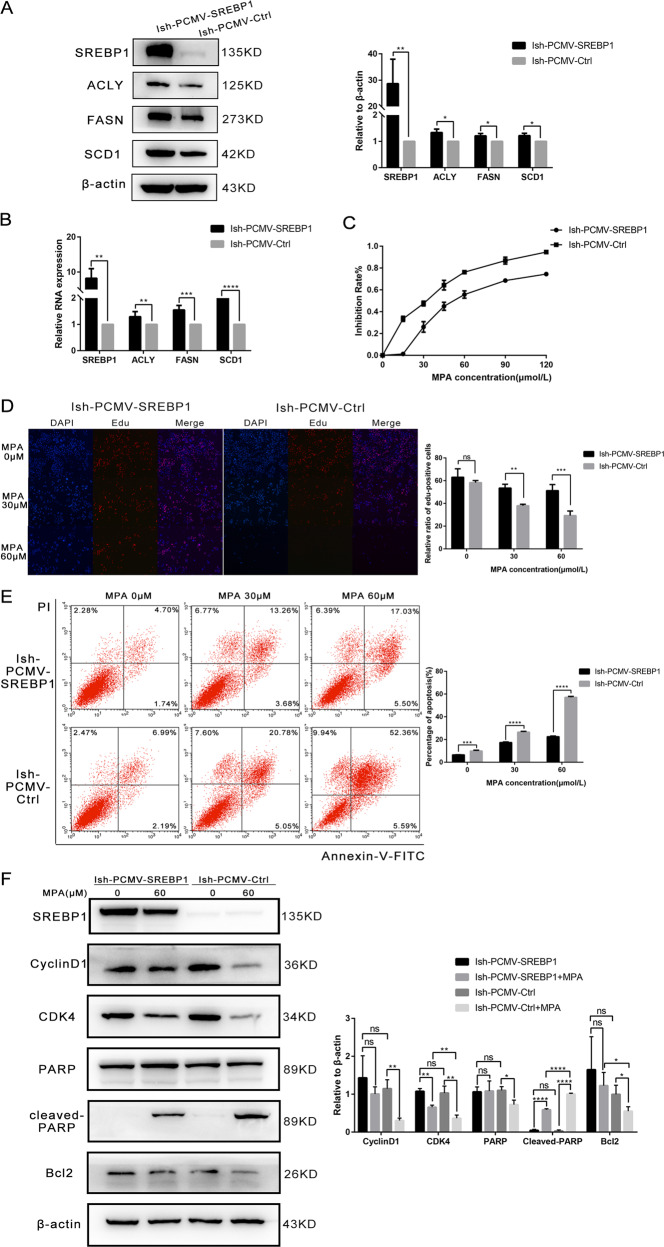

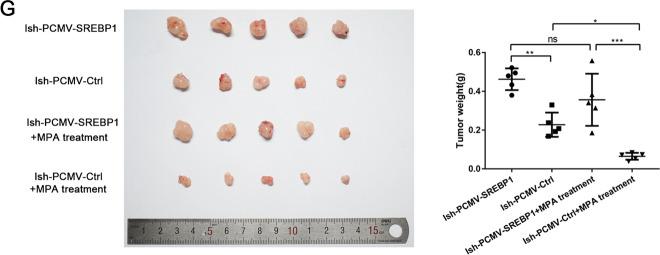
Changes of sensitivity of Ish cells to progesterone after overexpression of SREBP1. **A** Western blotting assay was used to detect the expression of SREBP1 and its target genes in PCMV-SREBP1 and PCMV-Ctrl Ish cells. **B** PCR assay was used to detect the mRNA expression of SREBP1 and its target genes in PCMV-SREBP1 and PCMV-Ctrl Ish cells. **C** Ish cells transfected with SREBP1 overexpression or negative control adenovirus were detected viability with different-dose MPA by MTT assay. **D** The proliferation capacity of PCMV-SREBP1 and PCMV-Ctrl Ish cells in MPA was demonstrated by EDU assay. **E** PCMV-SREBP1 and PCMV-Ctrl Ish cells were treated with 0.30.60 μM MPA for 48 h respectively. Apoptosis was detected by flow cytometry after staining with FITC Annexin-V and PI. Quantitive analysis of apoptotic ratio with CellQuest Pro software. **F** PCMV-SREBP1 and PCMV-Ctrl Ish cells were treated with 0.60 μM MPA for 48 h respectively. Expression of SREBP1, CylinD1, CDK4, Bcl-2, PARP, and cleaved PARP was determined by western blotting assay. 3 **G**. Images of tumors transfected with PCMV-SREBP1 or PCMV-Ctrl treated with MPA and the tumor weights of the four groups of mice were obtained from different treatments. All experiments were repeated three times at least. *P < 0.05, **P < 0.01, ***P < 0.001, and ****P < 0.0001 for statistical analysis of the indicated groups. Quantitation of western blotting assay bands shown in using Image J. Statistical analysis was performed using Student’s *t* test.

First, PCMV lentivirus was transfected into Ish cells to induce the overexpression of SREBP1. Western blotting and RT-PCR were then used to detect the expression of SREBP1 and its target genes, including ACLY, FASN, and SCD1, at the protein and mRNA levels (Fig. 3A, B). MTT assays showed that compared with PCMV-Ctrl cells, the IC_50_ of the PCMV-SREBP1 Ish cells almost doubled after 48-h of treatment with different concentrations of MPA. In addition, the PCMV-SREBP1 cells exhibited a stronger viability at the same concentration of MPA (Fig. 3C). EDU experiments were performed to detect the proliferation ability of PCMV-SREBP1 and PCMV-Ctrl cells after 48-h of incubation with different MPA concentrations (0, 30, and 60 μM). These assays showed that the proliferation ability of Ish-PCMV-SREBP1 cells was stronger at the same concentration of MPA than with the control cells (Fig. 3D). Furthermore, flow cytometry (FCM) showed that the proportion (%) of apoptotic cells in the Ish-PCMV-Ctrl group were higher than those in the Ish-PCMV-SREBP1 group after MPA treatment (Fig. 3E). Western blotting was then carried out to detect the expression of proteins related to proliferation and apoptosis; this was carried out in parallel with EDU experiments and FCM. Following MPA treatment, when compared with the Ish-PCMV-SREBP1 group, the expression levels of CDK4, CyclinD1, and Bcl2, in the Ish-PCMV-Ctrl group were significantly down-regulated while the expression of cleaved-PARP was significantly upregulated (Fig. 3F). Subsequently, we established nude-mouse xenograft tumor models using Ish-PCMV-SREBP1 and Ish-PCMV-Ctrl cells. When the mean tumor diameter reached 5 mm, we administered MPA (100 mg/kg/d) and an equal volume of normal saline, once every two days for 28 days. Results showed that MPA treatment showed no obvious inhibitory effect on the Ish-PCMV-SREBP1 tumors. However, in the Ish-PCMV-Ctrl group, MPA treatment was shown to significantly inhibit tumor growth (Fig. 3G).

**Fig. 4:**
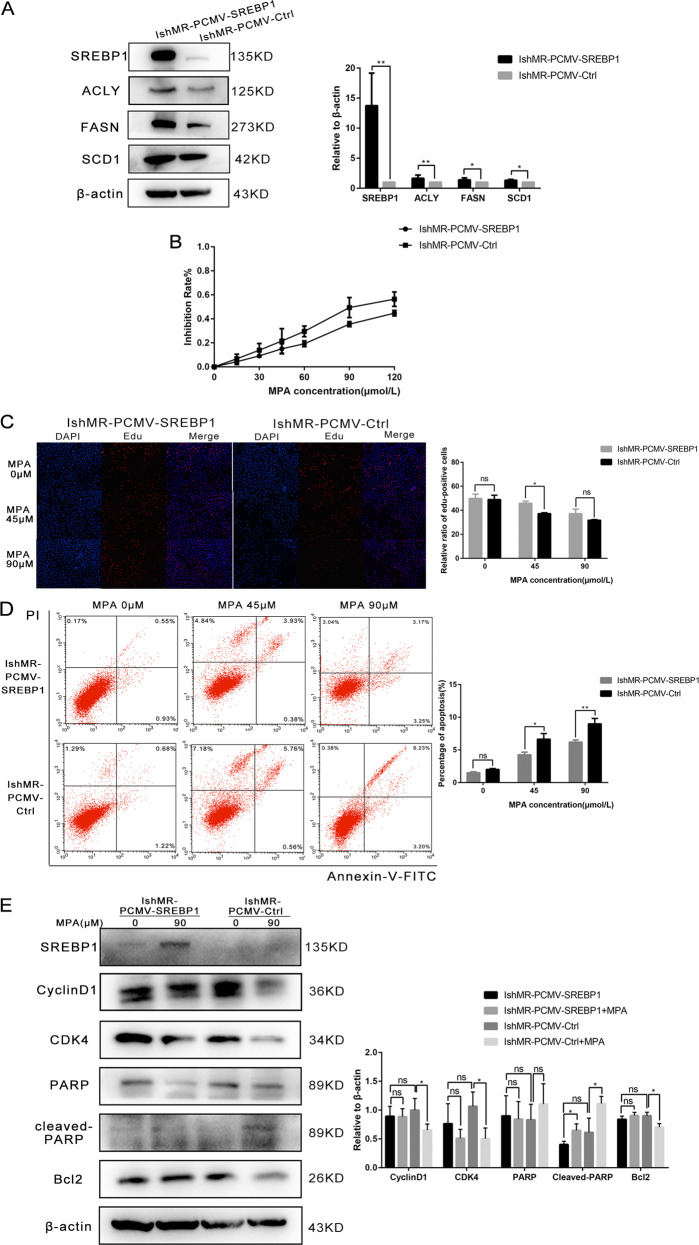
Changes of sensitivity of IshMR cells to progesterone after overexpression of SREBP1. **A** Western blotting assay was used to detect the expression of SREBP1 and its target genes in PCMV-SREBP1 and PCMV-Ctrl IshMR cells. **B** IshMR cells transfected with SREBP1 overexpression or negative control adenovirus were detected viability with different-dose MPA by MTT assay. **C** The proliferation capacity of PCMV-SREBP1 and PCMV-Ctrl IshMR cells in MPA were demonstrated by EDU assay. **D** PCMV-SREBP1 and PCMV-Ctrl IshMR cells were treated with 0.45.90 μM MPA for 48 h respectively. Apoptosis was detected by flow cytometry after staining with FITC Annexin-V and PI. Quantitive analysis of apoptotic ratio with CellQuest Pro software. **E** PCMV-SREBP1 and PCMV-Ctrl IshMR cells were treated with 0.90 μM MPA for 48 h respectively. Expression of SREBP1, CylinD1, CDK4, Bcl-2, PARP, and cleaved PARP were determined by western blotting assay. All experiments were repeated three times at least. *P < 0.05, **P < 0.01, ***P < 0.001, and ****P < 0.0001 for statistical analysis of the indicated groups. Quantitation of western blotting assay bands shown in using Image J. Statistical analysis was performed using Student’s *t* test.

PCMV lentivirus was transfected into IshMR cells to induce the overexpression of SREBP1. Western blotting and RT-PCR was then used to detect the mRNA and protein levels of SREBP1 and its target genes (Fig. 4A). MTT assays, EDU assays, and FCM showed that compared with PCMV-Ctrl cells, PCMV-SREBP1 cells exhibited a higher proliferative ability and were less vulnerable to apoptosis (Fig. 4B–D). Western blotting showed a similar trend (Fig. 4E).

Next, we compared functional tests following the overexpression of SREBP1 in Ish and IshMR cells. MTT and EDU assays showed that after 48 h of treatment with the same concentration of MPA, the viability and proliferative ability of the IshMR-PCMV-SREBP1 cells were significantly higher than for the Ish-PCMV-SREBP1 cells. Furthermore, the proportion of apoptosis in IshMR-PCMV-SREBP1 cells was significantly lower than in the Ish-PCMV-SREBP1 cells (Supplementary Fig. 1).

In summary, the overexpression of SREBP1 is capable of reducing the lethal effect of MPA on endometrioid adenocarcinoma cells and induces the emergence of progesterone resistance.

### The suppression of SREBP1 in progesterone-resistant cells inhibited cell proliferation, promoted apoptosis, and reversed progesterone resistance

**Fig. 5:**
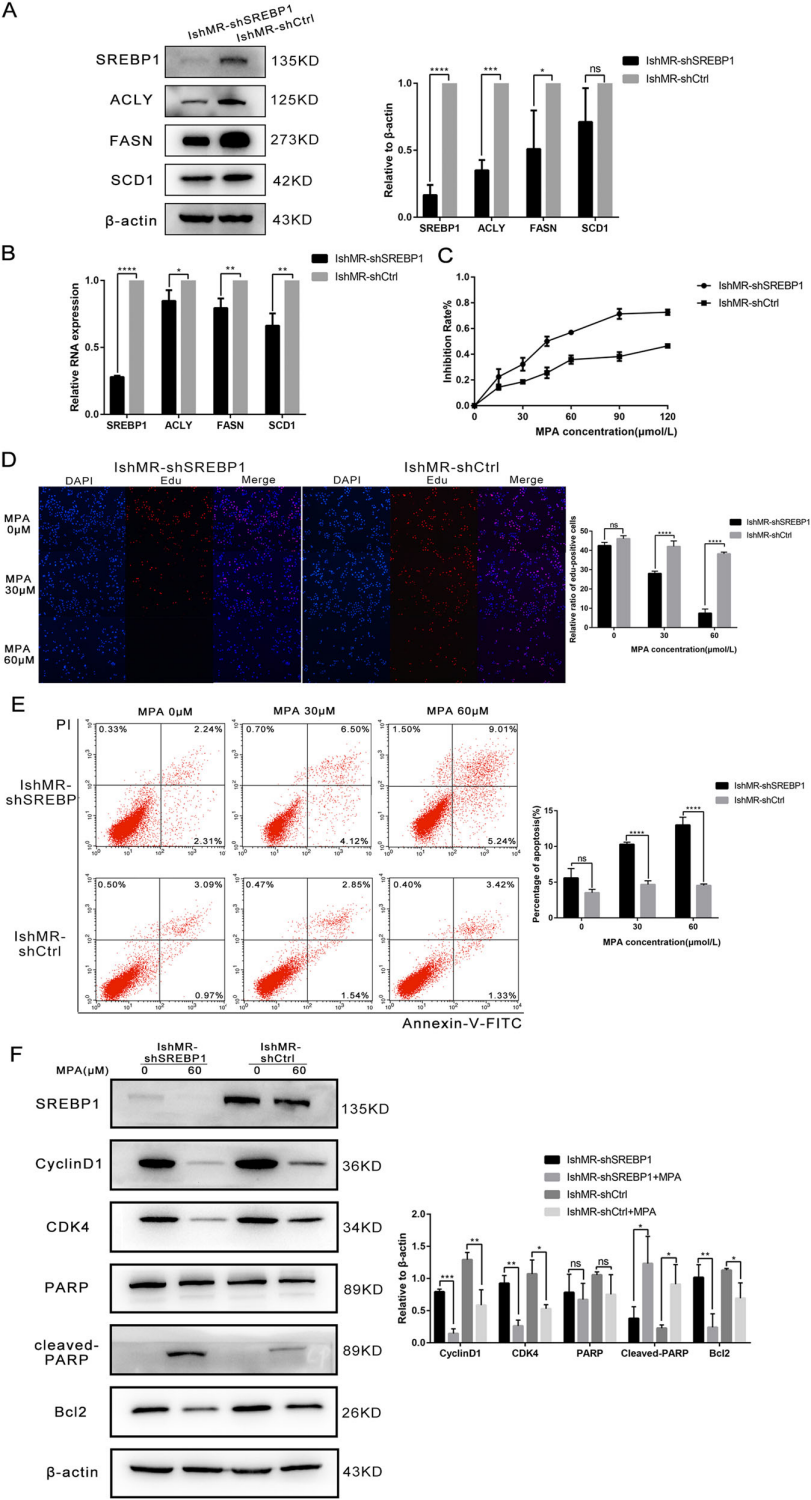

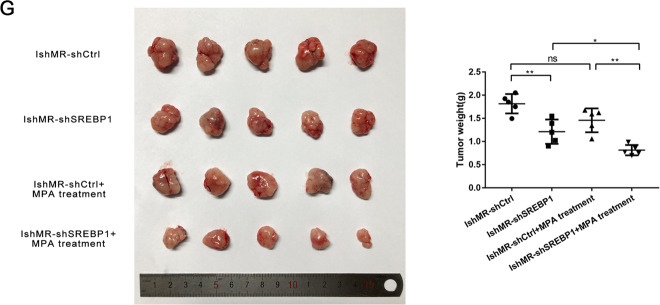
Changes of sensitivity of IshMR cells to progesterone after down expression of SREBP1. **A** Western blotting assay was used to detect the expression of SREBP1 and target genes in shSREBP1 and shCtrl IshMR cells. **B** PCR assay was used to detect the mRNA expression of SREBP1 and its target genes in shSREBP1 and shCtrl IshMR cells. **C** IshMR cells transfected with SREBP1 down expression or negative control adenovirus were detected viability with different-dose MPA by MTT assay. **D** The proliferation capacity of shSREBP1 and shCtrl IshMR cells in MPA were demonstrated by EDU assay. **E** shSREBP1 and shCtrl IshMR cells were treated with 0.30.60 μM MPA for 48 h respectively. Apoptosis was detected by flow cytometry after staining with FITC Annexin-V and PI. Quantitive analysis of apoptotic ratio with CellQuest Pro software. **F** shSREBP1 and shCtrl IshMR cells were treated with 0.60 μM MPA for 48 h respectively. Expression of SREBP1, CylinD1, CDK4, Bcl-2, PARP, and cleaved PARP were determined by western blotting assay. **G** Images of tumors transfected with shSREBP1 and shCtrl IshMR treated with MPA and the tumor weights of the four groups of mice were obtained from different treatments. All experiments were repeated three times at least. *P < 0.05, **P < 0.01, ***P < 0.001, and ****P < 0.0001 for statistical analysis of the indicated groups. Quantitation of western blotting assay bands shown in using Image J. Statistical analysis was performed using Student’s *t* test.

Next, we established IshMR-shSREBP1 cells by lentivirus transfection and investigated expression levels by western blotting and RT-PCR (Fig. 5A, B). MTT assays showed that the survival rates of IshMR-shSREBP1 cells after 48-hours of treatment with different MPA concentrations fell more significantly than those of the shCtrl group (Fig. 5C). EDU experiments were also carried out to investigate how MPA affected DNA synthesis. EDU assays showed that when compared with the control group, the DNA synthesis of IshMR-shSREBP1 cells declined sharply when treated with 0, 30, and 60 μM of MPA (Fig. 5D). FCM further revealed that IshMR-shSREBP1 cells were more sensitive to MPA than IshMR-shCtrl cells, and that the proportion of cells showing signs of apoptosis was significantly higher (Fig. 5E). The detection of proteins related to proliferation and apoptosis further confirmed these experimental findings (Fig. 5F). IshMR-shSREBP1 and IshMR-shCtrl nude-mouse xenograft tumor models were established and then treated with MPA. These experiments revealed that the IshMR-shSREBP1 group exhibited a greater extent of tumor shrinkage, while the IshMR-shCtrl group only exhibited a slight amount of shrinkage (Fig. 5G).

**Fig. 6:**
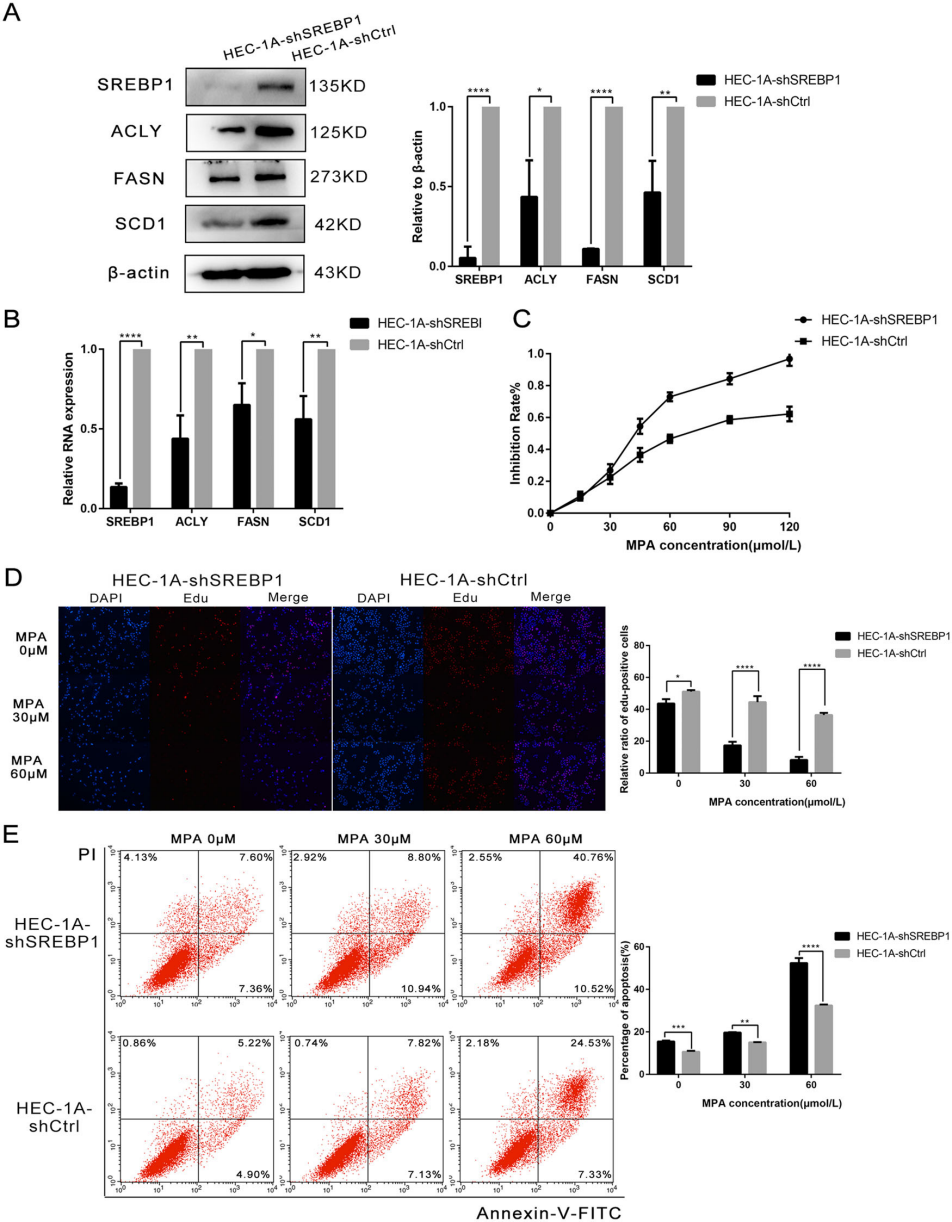
Changes of sensitivity of HEC-1A cells to progesterone after down expression of SREBP1. **A** Western blotting assay was used to detect the expression of SREBP1 and target genes in shSREBP1 and shCtrl HEC-1A cells. **B** PCR assay was used to detect the mRNA expression of SREBP1 and its target genes in shSREBP1 and shCtrl HEC-1A cells. **C** HEC-1A cells transfected with SREBP1 down expression or negative control adenovirus were detected viability with different-dose MPA by MTT assay. **D** The proliferation capacity of shSREBP1 and shCtrl HEC-1A cells in MPA were demonstrated by EDU assay. **E** shSREBP1 and shCtrl HEC-1A cells were treated with 0.30.60 μM MPA for 48 h respectively. Apoptosis was detected by flow cytometry after staining with FITC Annexin-V and PI. All experiments were repeated three times at least. *P < 0.05, **P < 0.01, ***P < 0.001, and ****P < 0.0001 for statistical analysis of the indicated groups. Quantitation of western blotting assay bands shown in using Image J. Statistical analysis was performed using Student’s *t* test.

In order to further demonstrate the role of SREBP1 in progesterone resistance, we next carried out experiments with primary progesterone-resistant HEC-1A cells. After verifying the knockout efficiency by WB and RT-PCR, we used MTT assays to determine the viability of HEC-1A-shSREBP1 and HEC-1A-shCtrl cells when treated with MPA. We also tested the proliferative capacity by EDU assays and determined the proportion of apoptotic cells by FCM. These results were all consistent with those derived from secondary progesterone-resistant cells (Fig. 6A–E).

In summary, the suppression of SREBP1 in progesterone-resistant cells enhanced the sensitivity of cells to progesterone and reversed progesterone resistance.

### The application of progesterone promoted the nuclear translocation of NF-κB and induced progesterone resistance via high expression levels of SREBP1

**Fig. 7:**
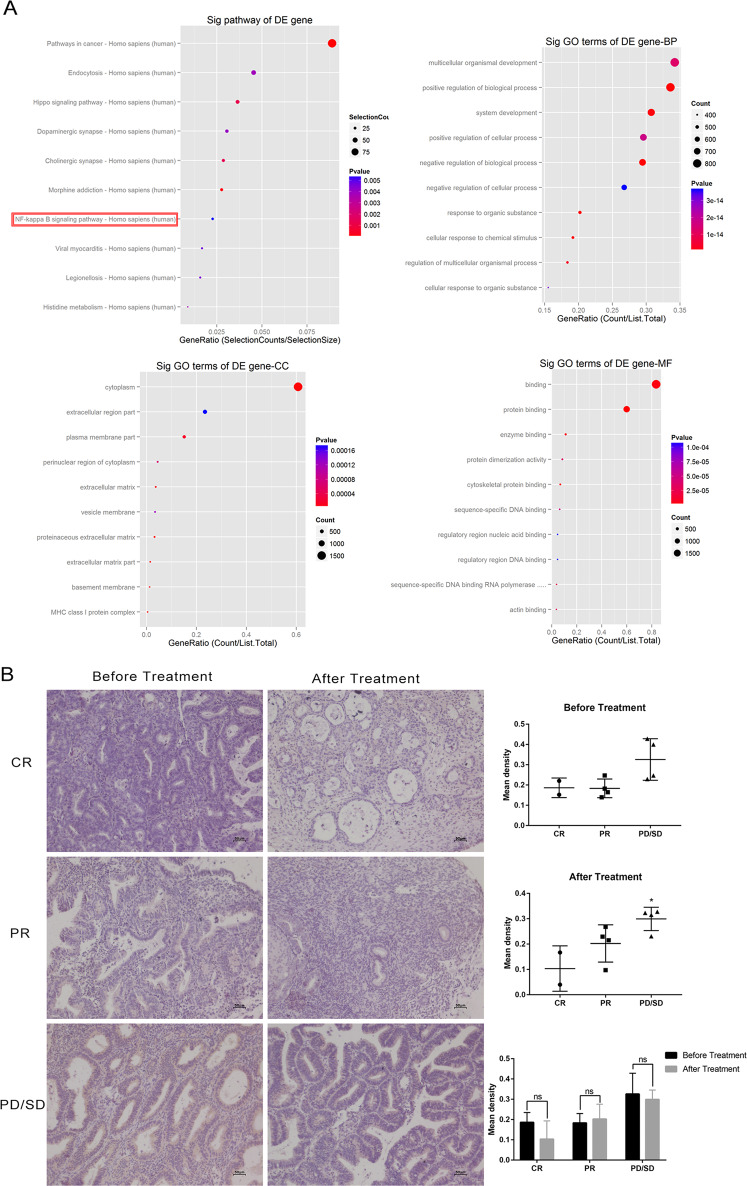

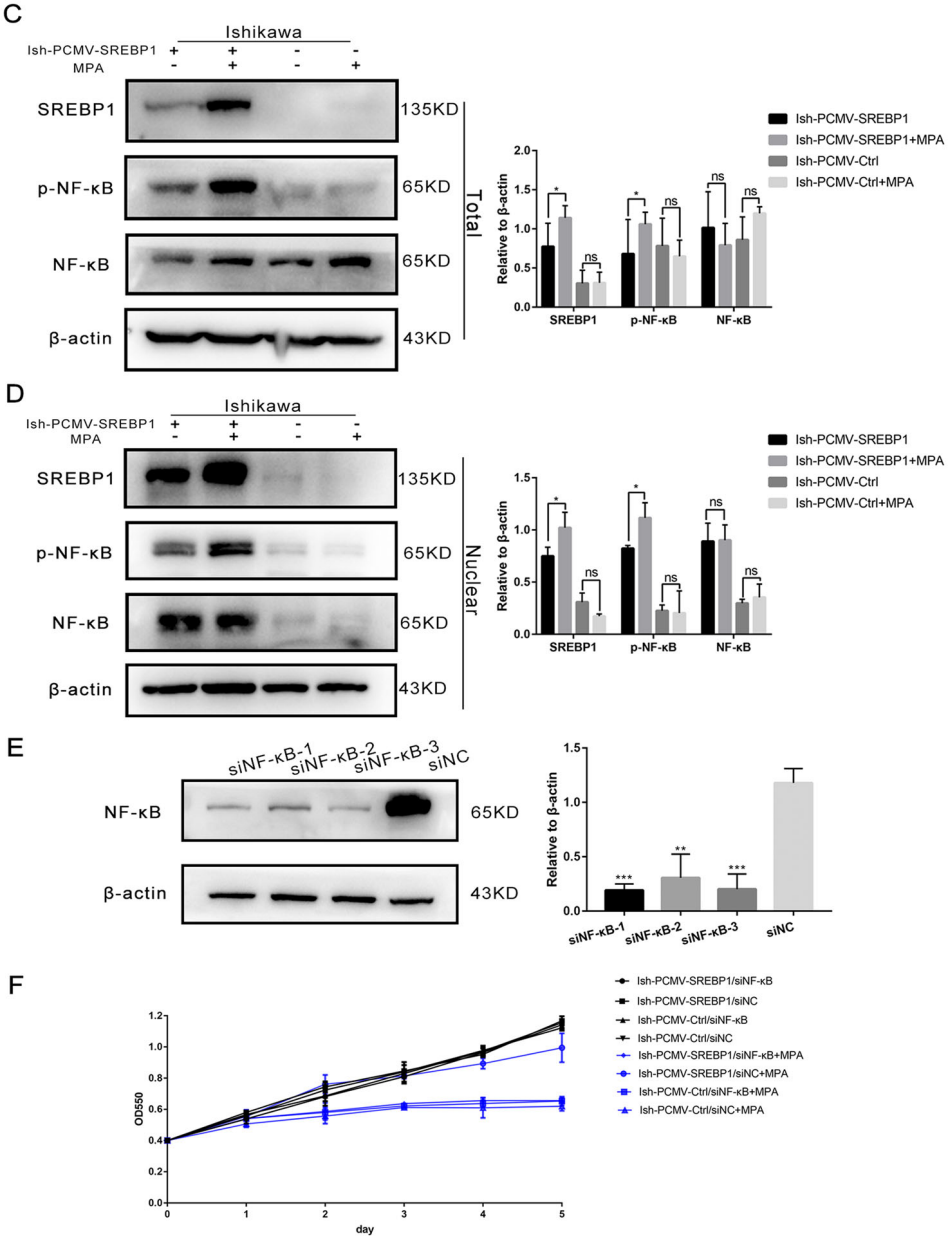

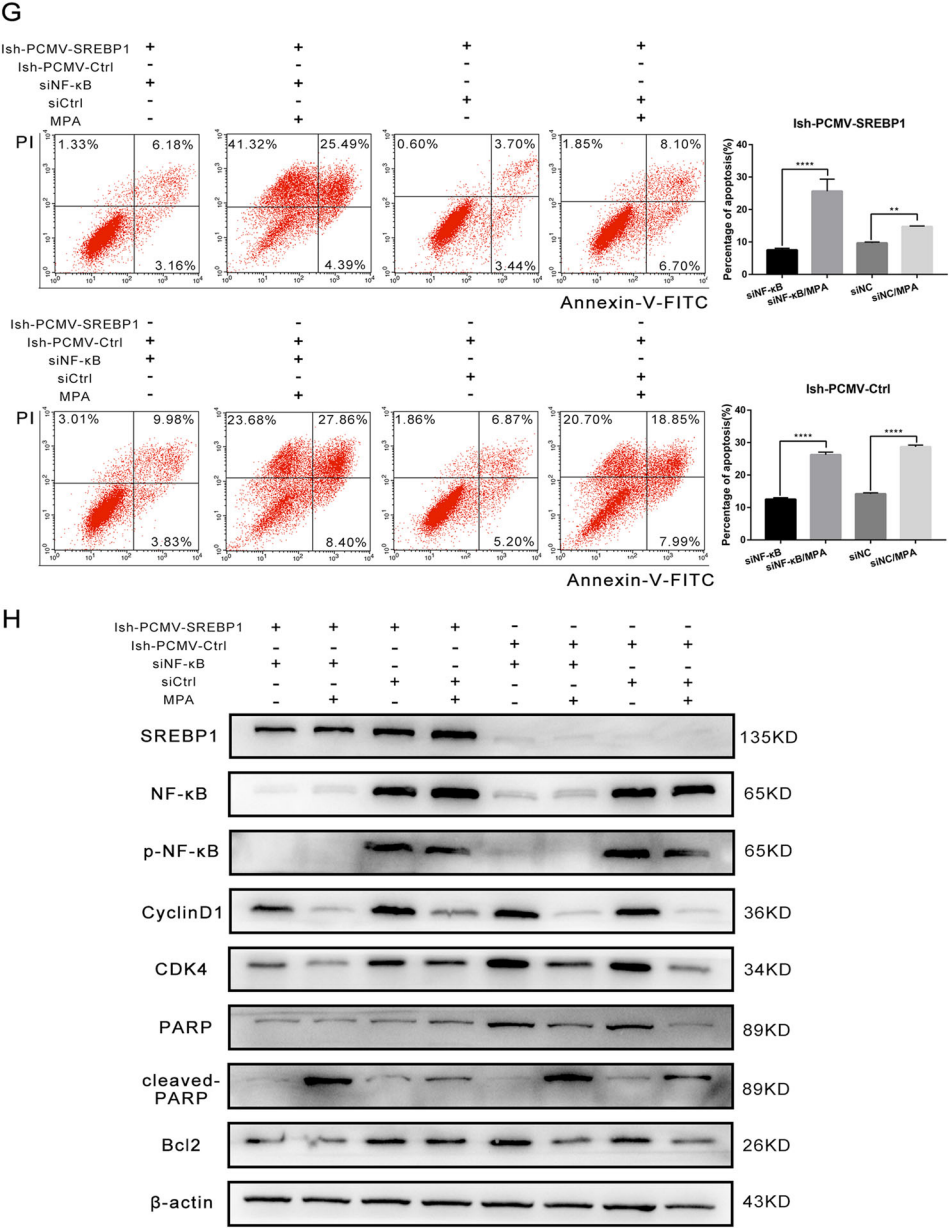
SREBP1 induces progesterone resistance by activating the NF-KB pathway. **A** The Top 10 signaling pathways in IshMR cell lines compared with Ish cell lines in KEGG pathway analysis; in GO-BP pathway analysis; GO-CC; GO-MF. **B** Representative images of IHC staining of NF*-*κB expression in endometrium before and after progesterone conservative treatment in CR, PR, and PD/SD group. The intensities of IHC staining were quantitated by Image-Pro Plus 6.0. **C** Effect of NF*-*κB pathway after overexpression of SREBP1 on MPA in Ish cells. **D** Effect of NF*-*κB pathway of nuclear expression after overexpression of SREBP1 on MPA in Ish cells. **E** Expression of NF*-*κB in PCMV-SREBP1 Ish cells were determined by western blotting assay after transfected with siNF*-*κB or siNC for 72 h. **F** Growth curves in PCMV-SREBP1 Ish cells and PCMV-Ctrl Ish cells with 30 μM MPA for 48 h were determined by MTT assay after transfected with siNF-κB or siNC. **G** Apoptosis in PCMV-SREBP1 Ish cells and PCMV-Ctrl Ish cells with 30 μM MPA for 48 h were determined by flow cytometry assay after transfected with siNF*-*κB or siNC. **H** PCMV-SREBP1 Ish cells and PCMV-Ctrl Ish cells with siNF-κB or siNC were treated with 30 μM MPA for 48 h respectively. Expression of SREBP1, NF-κB, p-NF*-*κB, CylinD1, CDK4, Bcl-2, PARP, and cleaved PARP were determined by western blotting assay. All experiments were repeated three times at least. *P < 0.05, **P < 0.01, ***P < 0.001, and ****P < 0.0001 for statistical analysis of the indicated groups. Quantitation of western blotting assay bands shown in using Image J. Statistical analysis was performed using Student’s *t* test.

To further explore the mechanism underlying the effect of SREBP1 on progesterone resistance in EC, we performed functional enrichment analyses for the upregulated gene via cluster using the Profiler R package, including gene ontology (GO) and Kyoto Encyclopedia of Genes and Genomes (KEGG) analyses. The results revealed pathway that the NF-κB pathway was enriched in the IshMR cell line (Fig. 7A). IHC was then used to stain NF-κB in clinical samples; this showed that NF-κB was highly expressed in progesterone-resistant samples (Fig. 7B). As shown in Fig. 7C, the overexpression of SREBP1 activated the NF-κB pathway and increased the expression levels of p-NF-κB. The expression of p-NF-κB increased significantly in Ish-PCMV-SREBP1 cells and Ish-PCMV-Ctrl cells following 24-h of MPA treatment. Western blotting further showed that the levels of nuclear NF-κB were significantly increased in Ish-PCMV-SREBP1 cells and that the content of NF-κB in the cytoplasm decreased after MPA treatment (Fig. 7D). These results indicate that SREBP1 induced progesterone resistance by promoting the translocation of NF-κB to the nucleus and by activating the NF-κB pathway.

To confirm these conclusions, we knocked down NF-κB in Ish-PCMV-SREBP1 cells by siRNA and then carried out rescue experiments to detect the drug resistance properties of these cells. First, we identified two siRNA sequences in Ish cells for which the knockout efficiency exceeded 80%; we selected one of these sequences for subsequent experiments (Fig. 7E). Ish-PCMV-SREBP1 cells and Ish-PCMV-Ctrl cells were transfected with siNF-κB and siNC for 24 h and then seeded into 96-well plates. Then different concentrations MPA were added into medium, and it was found that knocking out NF-κB in PCMV-Ctrl Ish cells help them regain sensitivity to MPA, accordingly, their viability decreased (Fig. 7F), and apoptosis proportion rised (Fig. 7G). Subsequent apoptosis-related proteins detection in the way of Western-blot illustrated that NF-κB gene knockout were capable of reversing the changes of Bcl2, CDK4, and other genes which were induced by SREBP1 overexpression (Fig. 7H).

To draw a conclusion, the endometrial cancer cells are sensitive to progesterone, after knocking out NF-κB gene in the cells which are progesterone-resistant through overexpression of SREBP1. In the meantime, we declared that SREBP1 participates in progesterone resistance through activating the NF-κB pathway.

### Fatostatin reversed progesterone resistance by inhibiting the expression of SREBP1

**Fig. 8:**
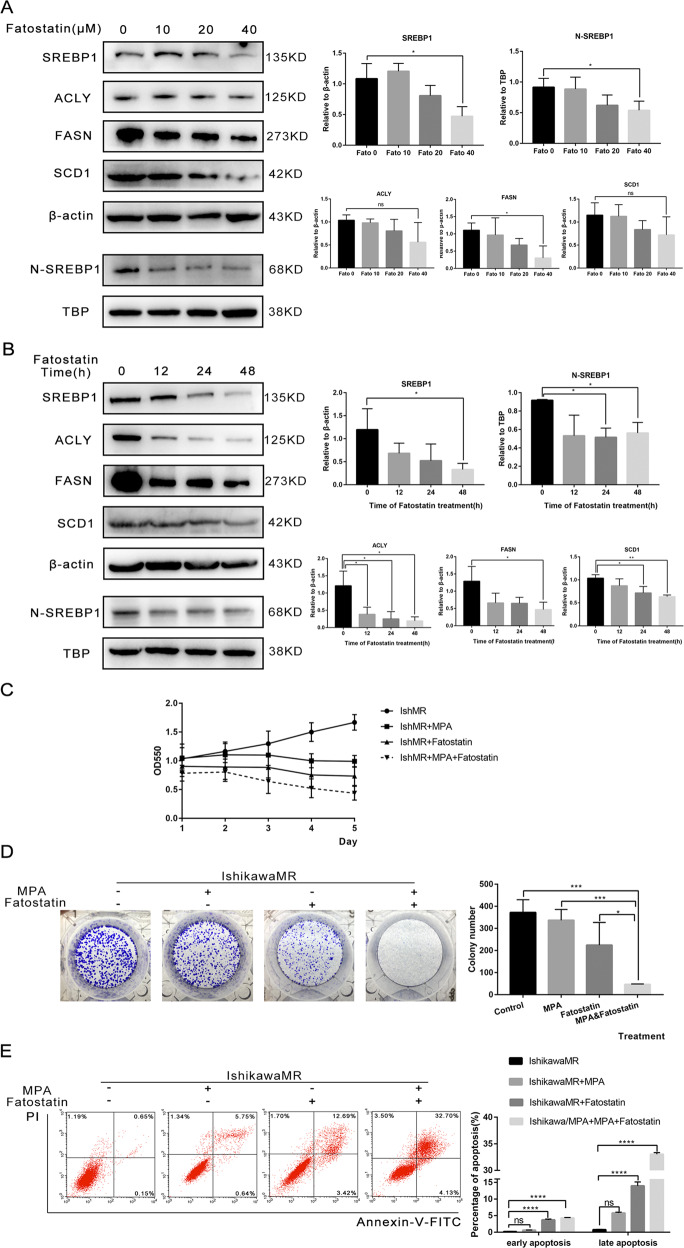

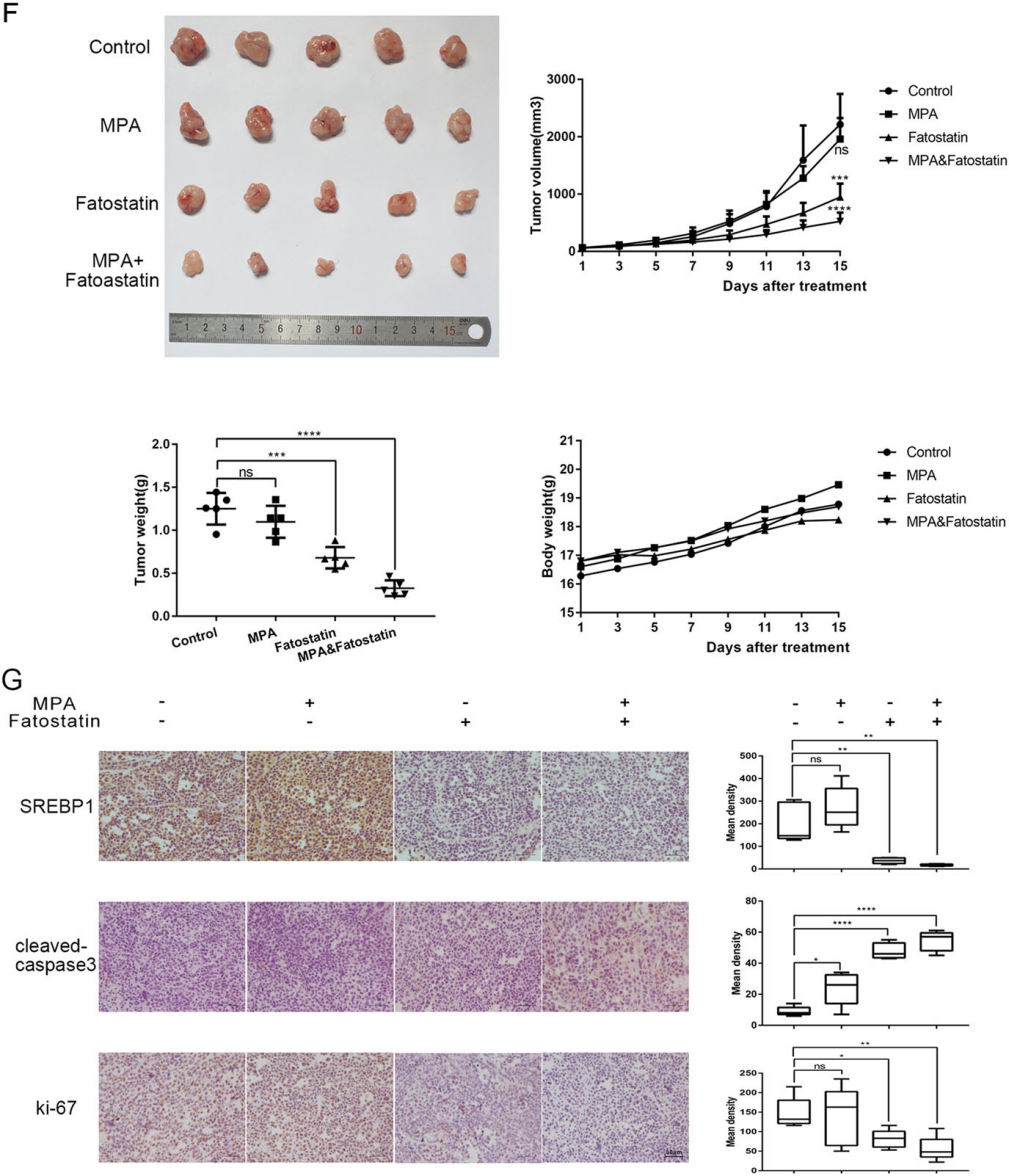
The sensitization effect of Fatostatin on MPA in vivo and vitro. **A**, **B** Expression of SREBP1 and target genes inhibited by Fatostatin in a dose-dependent and time-dependent manner. **C** The effects of MPA (30 μM) and Fatostatin (20 μM) treatment on cell viability detected by the MTT assay. **D** The effects of MPA (30 μM) and Fatostatin (20 μM) treatment on the colony- forming ability. **E** The effects of MPA (30 μM) and Fatostatin (20 μM) treatment on apoptosis of IshMR cells detected by flow cytometry. **F** Images of tumors of IshMR cells with MPA, Fatostatin and combined drug. And the volume and weight of tumor and the body weight of the four groups of mice. **G** Representative images of IHC staining of SREBP1, cleaved-caspase-3 and Ki-67 in tumor tissues. All experiments were repeated three times at least. *P < 0.05, **P < 0.01, ***P < 0.001, and ****P < 0.0001 for statistical analysis of the indicated groups. Quantitation of western blotting assay bands shown in using Image J. Statistical analysis was performed using Student’s *t* test.

Previous studies have shown that Fatostatin inhibits the proliferation of endometrioid adenocarcinoma cells, and enhances apoptosis, by blocking the metabolic pathway regulated by SREBP1. In light of the important role that SREBP1 plays in progesterone resistance, we hypothesized that the use of Fatostatin to target SREBP1 may have a significant therapeutic effect. As shown in Fig. 8A, B, Fatostatin inhibited the expression of SREBP1, and its target genes, in both a dose- and time-dependent manner. MTT analysis indicated that the treatment of IshMR cells with 30 μM MPA alone exerted no obvious inhibitory effect over the course of 1–5 days. There was no clear inhibitory effect following treatment with 20 μM Fatostatin; however, the combination of Fatostatin and MPA treatment did result in a significant reduction in cell viability (Fig. 8C). To confirm the long-term effects of these two drugs on progesterone-resistant cell proliferation, we used a colony formation method; results were consistent with those derived from the MTT assay (Fig. 8D). Further experiments revealed that the combination of Fatostatin and progesterone significantly promoted cell apoptosis when comparison with the control group, MPA group, and the Fatostatin group (Fig. 8E). Together, these data illustrate that Fatostatin increases the sensitivity of EC cells to progesterone.

### Co-treatment with Fatostatin and MPA synergistically inhibited the proliferation of progesterone-resistant EC cells in vivo

To further verify the sensitization effect of Fatostatin combined with progesterone, 10^7^ IshMR cells were injected into the armpits of 4-week-old nude female mice. When the diameter of tumors reached ~5 mm, the mice were randomly divided into four groups (five in each group) for 2-week treatments; the injection of normal saline every day, the injection of MPA (100 mg/kg) or Fatostatin once every two days, and the alternate injection of MPA (100 mg/kg) or Fatostatin every other day. Tumor diameter and body weight were measured every two days to monitor the effect of drug treatment. Results showed that the combined treatment significantly inhibited tumor growth when compared with the control group and the groups treated with single agents. There were no significant differences between the groups in terms of body weight (Fig. 8F). IHC was used to stain tumors for SREBP1, cleaved-casepase3, and ki-67 and showed that expression of SREBP1 and ki-67 were lowest and expression of cleaved-caspase3were highest in the combined treatment group (Fig. 8G). Consequently, when applied in vivo, Fatostatin enhanced the sensitivity of endometrioid adenocarcinoma to progesterone, thus inhibiting tumor growth.

## Discussion

Endometrial cancer ranks as the fifth most common form of gynecological cancer among women in developed countries, with an estimated 382, 069 new cases diagnosed worldwide each year^26^. Although EC is generally identified as a postmenopausal cancer, 14–25% of patients are diagnosed during the premenopausal period. Approximately 5% of patients are younger than 40 years of age^27^; these patients are strongly inclined to adopt conservative medical treatments, including fertility-sparing treatments such as progesterone, medroxyprogesterone acetate, or GnRH. However, conservative treatments also carry risks; for instance, these treatments might fail or lead to recurrence. Research has shown that the primary cause of these risks in patients with EC is the development of progesterone resistance. Currently, women of reproductive age are inclined to delay childbearing; consequently, the incidence of EC among nulliparous women is increasing. As such, there is an urgent need to investigate the mechanisms involved with progesterone resistance in patients with EC. Over the past few years, several studies have attempted to investigate the mechanisms underlying progesterone resistance^16–19,25,28–34^. It is widely believed that the PGR is a prerequisite for progesterone response. Furthermore, the continuous delivery of progesterone is capable of reducing the expression levels of the PR; this leads to progesterone desensitization and drug resistance^35,36^. In addition, other molecules may also contribute to progesterone resistance, including the PI3K-Akt, EMT, and insulin signaling pathways.

SREBPs are transcription factors that regulate the anabolism of cholesterol and lipids. Specifically, SREBPs activate the transcription of their target genes by binding with the sterol regulatory elements that exist in the promoter or enhancer of the lipid synthase gene. It has also been found that SREBP1 is involved in the genesis and development of multiple human tumors including EC^6–10,16,37,38^ although the precise mechanisms associated with the progesterone resistance of EC has yet to be elucidated. According to our research, the expression levels of SREBP1 in the tissues of patients who have acquired progesterone resistance are higher than those detected in patients who have fully recovered from progesterone treatment. The resistance of selected clinical samples to progesterone can be verified by staining the PGR.

In order to fully understand and explore the molecular mechanisms underlying the role of SREBP1 in progesterone resistance, we established stable progesterone-resistant cell lines. Next, we compared primary and secondary progesterone-resistant cells together with progesterone-sensitive cells. This allowed us to confirm whether SREBP1 participates in the process of progesterone resistance or not. In the present study, we found that the over-expression of SREBP1 was able to promote the viability of Ish and IshMR cells and increase their resistance to progesterone, especially in cells that were sensitive to progesterone. In contrast, IshMR-shSREBP1 caused IshMR cells to become sensitive to progesterone and suppressed their proliferation. Data derived from our in vivo assays were consistent with those derived from in vitro assays. Similar findings have been reported for breast cancer, pancreatic cancer, and hepatocellular carcinoma. In other words, there is strong evidence to support the fact that SREBP1 is involved in tumor resistance and can be used as a predictor for chemotherapy resistance^27,28,39^. Furthermore, research carried out thus far indicates that SREBP1 is not only able to promote the progression of EC, but could also participate during the formation of progesterone resistance in EC.

Previous research has demonstrated that SREBP1 participates in the occurrence and development of colorectal cancer and renal clear cell carcinoma and does so by regulating the NF-κB pathway^18,19^. This suggests that we should investigate whether SREBP1 regulates the NF-κB pathway in cases of progesterone resistance or not. The existing literature shows that NF-κB forms an inactive complex in combination with inhibitory cytoplasmic proteins. When stimuli are received from the extracellular environment, NF*-*κB breaks away from the inactive complex, translocates to the nucleus, makes contact with DNA and activates downstream pathways, thus leading to functional changes and the activation or suppression of specific genes. In our present study, we found that the overexpression of SREBP1 was able to promote the nuclear translocation and activation of the NF-κB pathway; this could increase the expression levels of phosphorylated NF-κB. Furthermore, this effect is likely to be stronger in cells that were treated with progesterone. To further verify this hypothesis, we carried out rescue experiments in which we knocked out NF-κB to inhibit activation in Ish-PCMV-SREBP1 cell lines. However, we did not observe any resistance to progesterone in these cells. Together, these data indicate that SREBP1 plays a role in progesterone resistance in patients with EC and that this effect involves the activation of the NF-κB pathway.

Fatostatin is a specific inhibitor of SREBP1 and is known to exert effects on prostate cancer and endometrial cancer^23–25^. However, the effects of Fatostatin on progesterone resistance in EC has still to be fully elucidated. Fatostatin is not only able to block the formation of lipids in response to insulin stimulation, but is also capable of reducing total fatty acids, triglycerides, and low-density lipoproteins, as well as body weight, by interfering with the intracellular transcription of SREBP1^29^. Our study is the first to demonstrate that Fatostatin reverses progesterone resistance in EC and enhances progesterone sensitization; these effects occurred both in vivo and in vitro by inhibiting the expression of SREBP1. Furthermore, the combination of Fatostatin and progesterone could be adopted clinically to improve the sensitivity and efficacy of patients who receive conservative treatments for EC.

In summary, this is the first study to confirm that SREBP1 is one of the molecular factors involved in progesterone resistance in endometrial cancer and acts via the NF-κB pathway. We hypothesize that high expression levels of SREBP1 could be considered as a standard biomarker to evaluate the efficacy of progesterone treatment in cases of EC. Our data also demonstrate that the application of Fatostatin, an inhibitor of SREBP1, can reverse progesterone resistance in EC patients, and can help to treat patients with endometrial cancer, especially those receiving conservative treatments.

## Materials and methods

### Tissue samples and immunohistochemistry assays

We acquired tissues from 17 patients who underwent treatment at Qilu Hospital of Shandong University between 2010 and 2017. The tissues were collected from the Pathology Department at Qilu Hospital. These patients did not have any other diseases of the reproductive system. The pathological diagnosis of EC or hyperplasia was made in accordance with the latest National Comprehensive Cancer Network (NCCN) guidelines. All patients received medroxyprogesterone acetate for at least 6 months and were followed up regularly. A CR was defined as the absence of residual hyperplasia or cancer in more than 95% of the tissue. A PR was defined as <50% of residual hyperplastic glands. If more than 50% of residual hyperplasia was evident, and the extent of hyperplasia was similar to or worse than before progesterone treatment, then the patients were classified as PD or SD^40–42^.

All human endometrial tissue samples were dehydrated for 1 h and dewaxed with xylene and ethyl alcohol. We then used a microwave antigen retrieval technique to repair the antigen. We then stained the tissues antibodies against SREBP1(1:200), PGR(1:50), NF-κB(1:200), ki67(1:500) and cleaved-casepase3(1:800). Positive staining was subsequently visualized with 3,3′-diaminobenzidine (DAB) and counterstained with hematoxylin Detailed experimental and analytical methods for IHC were described previously^30^.

### Antibodies and agents

For this study, we purchased the following antibodies against SREBP1 (Abcam, ab191857), FASN(Abcam,ab96866), ACLY(Abcam,ab40793), SCD1(Abcam,ab236868), PGR (Abcam, ab32085), Cyclin D1 (Cell Signaling Technologies, #2978), CDK4 (Cell Signaling Technologies, #12790), PARP (Cell Signaling Technologies, #9542), cleaved-PARP (Cell Signaling Technologies, #5625), Bax (Cell Signaling Technologies, #2772), Bcl2 (Cell Signaling Technologies, #4223), NF-κB(Cell Signaling Technologies,#8242), p-NF-κB (Cell Signaling Technologies, #3033), β-actin (Cell Signaling Technologies, #4970), ki67 (Abcam, ab92742), cleaved-casepase3 (Cell Signaling Technologies, #9664), mouse IgG (Cell Signaling Technologies, #7076), and rabbit IgG (Cell Signaling Technologies, #7074). Medroxyprogesterone acetate and Fatostatin were obtained from Abcam and MedChem Express, respectively, and were both diluted in DMSO.

### Cell culture

Ishikawa cells (referred to throughout this paper as ‘Ish’) and HEC-1A cells were purchased from Shanghai Zhong Qiao Xin Zhou Biotechnology Co. MPA was used to establish an MPA-resistant Ish cell line which we referred to as IshikawaMR (or ‘IshMR’); this cell line was created via the increasing concentration gradient method^31^. Ish and IshMR cells were cultured in RPMI1640 medium (BI, USA) containing 10% fetal bovine serum (BI, USA) at 37 °C in a 5% CO_2_ humidified atmosphere. HEC cells were routinely grown in M5A media at 37 °C in a 5% CO_2_ humidified atmosphere. We also 10 μM MPA was added to the medium containing the IshMR cells to maintain resistance.

### MTT assays

MTT assays were used to analyze cell viability and determine the 50% inhibitory concentration (IC_50_); 0.3 × 10^4^ cells were seeded into each well on a 96-well plate and cultured overnight. Then, the cells were treated with either the vehicle control or different concentrations of MPA (15, 30, 45, 60, 90, and 120 μM) for 48 h. Then, 10 μL of MTT (5 mg/mL in PBS) was added to each well at 37 °C for 4 h. Formazan crystals were dissolved in 150 μL of dimethylsulfoxide (DMSO; Sigma-Aldrich, St Louis, MO, USA). Absorbance was then detected at a wavelength of 550 nm.

### EDU incorporation assays

We used a 5-ethynyl-20-deoxyuridine (EDU) incorporation assay kit (Ribobio, Guangzhou, China) to test the proliferative ability of cells; this kit was used in accordance with the manufacturer’s instructions. In total, 0.6 × 10^4^ cells were seeded into each well of a 96-well plate per well and cultured overnight; then, fresh medium was added containing specific concentrations of MPA (0, 30, and 60 μM or 0, 45, and 90 μM). After 48 h, 50 μM of EDU was added to fix and stain the cells. Finally, cell nuclei were stained with 1 × Hoechst and detected by fluorescence microscopy.

### Evaluating cellular apoptosis by FCM

After treatment with either control media or different concentrations of MPA (30 and 60 μM) for 48 h, we collected 6 × 10^4^ cells; these were then stained, gently vortexed, and incubated for 15 min at normal temperature. Then, we performed a cell apoptosis assay using a FACS flow cytometer and a FITC Annexin V Apoptosis Detection Kit (BD Bioscience Pharmingen, San Diego, CA, USA); the kit was used in accordance with the manufacturer’s instructions. Finally, data were analyzed by Cell Quest software (Becton Dickinson, Franklin Lakes, NJ, USA).

### Western blot analysis

Cells were lysed in a mixed buffer that contained RIPA, NaF, and PMSF. Protein concentrations were analyzed using a BCA protein assay kit (Tiangen Biotech Co., Ltd., Beijing, China). Protein was resolved by 10% SDS-PAGE and transferred to PVDF membranes (Millipore, Bedford, MA, USA). Membranes were incubated overnight with the indicated primary antibodies at 4 °C and then incubated with appropriate secondary antibodies for 2 h at room temperature. Protein bands were detected by ImageQuant LAS4000 (General Electric Company, Boston, MA, USA) and quantified by ImageJ software. β-actin was detected as a loading control.

### Quantitative real‑time transcription‑polymerase chain reaction

Total RNA was extracted from cells and the concentration and purity was evaluated with a spectrophotometer (Thermo Fisher Scientific Inc., MA, USA). RNA was then reverse transcribed into cDNA (3000 ng/10 μl reaction system). PCR reactions were then performed on a StepOne™ PCR amplifier (Applied Biosystems, USA) with SYBR-green (TAKARA, Japan) in a 10 μl reaction system; β-actin was used as a control. The primers used are shown in the Supplementary information(Supplement Table 1).

### Xenograft model

The female BALB/c mice used in the present study were treated in accordance with protocols approved by the ethical committee of Shandong University. 1 × 10^7^ cells were mixed with 100 μl of PBS and injected subcutaneously into the right armpit of each 5-week-old nude female mice. When the tumor diameter reached 5 mm, the mice were then randomly divided into different groups (*n* = 5 mice group) to receive an intraperitoneal injection of either vehicle (control), MPA (100 mg/kg/bodyweight), Fatostatin (25 mg/kg/bodyweight) or MPA plus Fatostatin. The MPA was injected once every two days and Fatostatin was injected three times per week. The control mice were injected with the same volume of plasmid only. The mice were killed on day 28. Each day, we measured tumor size with calipers and calculated the tumor volume as follows: tumor volume = width^2^ × length/2. We also determined bodyweight each day to monitor for side-effects.

### Separation of the nucleus from cytoplasm

A Nucleoprotein Extraction Kit (BD) was used to separate cell nuclei from the cytoplasm. The cells were washed three times with cold PBS and then centrifuged at 500 g for 5 min at 4 ˚C; then, the supernatants were discarded. Cell lysates were added to the precipitates and vortexed for 15 s. Then, the lysates were placed on ice and shaken for 20 min. Subsequently, the suspensions were centrifuged for 5 min at 12,000 × *g*; at this point, the supernatants contained cytoplasmic proteins. The nuclear lysates were present in the precipitates. Lysis was performed by vortexing for 15 s every 10 min for 40 min. The suspensions were then centrifuged for 10 min at 12,000 × *g*; at this point, the supernatants contained the nuclear proteins.

### Colony formation assay

Six hundred IshMR cells were seeded into each well of a six-well plate and cultured overnight; the medium was then replaced with a medium containing different drugs. Cells were incubated with the drugs at 37 °C for 14 day, fixed with 4% paraformaldehyde for 15 min, and then stained with crystal violet (Beyotime, Beijing, China) for 30 min.

### Statistical analysis

All experiments were repeated at least three times. Data were analyzed using GraphPad Version 7.0 software and expressed as mean ± standard deviation (SD). Statistical significance was determined by Student’s *t* tests, one-way analysis of variance (ANOVA), and two-way ANOVA. Statistical significance was set at *P* < 0.05.

## Supplementary information

supplement figure legends

supplement figure 1

supplement Table 1

## References

[1] Siegel RL, Miller KD, Jemal A (2020). Cancer statistics, 2020. CA.

[2] Ushijima K (2007). Multicenter phase II study of fertility-sparing treatment with medroxyprogesterone acetate for endometrial carcinoma and atypical hyperplasia in young women. J. Clin. Oncol..

[3] Sorosky JI (2012). Endometrial cancer. Obstet. Gynecol..

[4] Kaku T (2001). Conservative therapy for adenocarcinoma and atypical endometrial hyperplasia of the endometrium in young women: central pathologic review and treatment outcome. Cancer Lett..

[5] Ramirez PT, Frumovitz M, Bodurka DC, Sun CC, Levenback C (2004). Hormonal therapy for the management of grade 1 endometrial adenocarcinoma: a literature review. Gynecol. Oncol..

[6] Brown MS, Goldstein JL (1997). The SREBP pathway: regulation of cholesterol metabolism by proteolysis of a membrane-bound transcription factor. Cell.

[7] Witkowski A, Rangan VS, Randhawa ZI, Amy CM, Smith S (1991). Structural organization of the multifunctional animal fatty-acid synthase. Eur. J. Biochem..

[8] Huang WC, Li X, Liu J, Lin J, Chung LW (2012). Activation of androgen receptor, lipogenesis, and oxidative stress converged by SREBP-1 is responsible for regulating growth and progression of prostate cancer cells. Mol. Cancer Res..

[9] Heemers H, Vanderhoydonc F, Heyns W, Verhoeven G, Swinnen JV (2000). Progestins and androgens increase expression of Spot 14 in T47-D breast tumor cells. Biochem. Biophys. Res. Commun..

[10] Yin F (2017). TIP30 regulates lipid metabolism in hepatocellular carcinoma by regulating SREBP1 through the Akt/mTOR signaling pathway. Oncogenesis.

[11] Cai HL (2015). A potential mechanism underlying atypical antipsychotics-induced lipid disturbances. Transl. Psychiatry.

[12] Shimano H (2001). Sterol regulatory element-binding proteins (SREBPs): transcriptional regulators of lipid synthetic genes. Prog. Lipid Res..

[13] Walker AK (2011). A conserved SREBP-1/phosphatidylcholine feedback circuit regulates lipogenesis in metazoans. Cell.

[14] Dong XY, Tang SQ (2010). Insulin-induced gene: a new regulator in lipid metabolism. Peptides.

[15] Kowalik MK, Slonina D, Rekawiecki R, Kotwica J (2013). Expression of progesterone receptor membrane component (PGRMC) 1 and 2, serpine mRNA binding protein 1 (SERBP1) and nuclear progesterone receptor (PGR) in the bovine endometrium during the estrous cycle and the first trimester of pregnancy. Reprod. Biol..

[16] Li W (2012). Repression of endometrial tumor growth by targeting SREBP1 and lipogenesis. Cell Cycle.

[17] Qiu C, Dongol S, Lv QT, Gao X, Jiang J (2013). Sterol regulatory element-binding protein-1/fatty acid synthase involvement in proliferation inhibition and apoptosis promotion induced by progesterone in endometrial cancer. Int. J. Gynecol. Cancer.

[18] Gao Y (2019). SREBP1 promotes the invasion of colorectal cancer accompanied upregulation of MMP7 expression and NF-kappaB pathway activation. BMC Cancer.

[19] Yang H (2018). SREBP1-driven lipid desaturation supports clear cell renal cell carcinoma growth through regulation of NF-kappaB signaling. Biochem. Biophys. Res. Commun..

[20] Choi Y, Kawazoe Y, Murakami K, Misawa H, Uesugi M (2003). Identification of bioactive molecules by adipogenesis profiling of organic compounds. J. Biol. Chem..

[21] Kamisuki S (2009). A small molecule that blocks fat synthesis by inhibiting the activation of SREBP. Chem. Biol..

[22] Shao W, Machamer CE, Espenshade PJ (2016). Fatostatin blocks ER exit of SCAP but inhibits cell growth in a SCAP-independent manner. J. Lipid Res..

[23] Gao S (2018). Fatostatin suppresses growth and enhances apoptosis by blocking SREBP-regulated metabolic pathways in endometrial carcinoma. Oncol. Rep..

[24] Li X, Chen YT, Hu P, Huang WC (2014). Fatostatin displays high antitumor activity in prostate cancer by blocking SREBP-regulated metabolic pathways and androgen receptor signaling. Mol. Cancer Ther..

[25] Brovkovych V (2018). Fatostatin induces pro- and anti-apoptotic lipid accumulation in breast cancer. Oncogenesis.

[26] Flores VA, Vanhie A, Dang T, Taylor HS (2018). Progesterone receptor status predicts response to progestin therapy in endometriosis. J. Clin. Endocrinol. Metab..

[27] Zhou C (2019). Resveratrol enhances the chemotherapeutic response and reverses the stemness induced by gemcitabine in pancreatic cancer cells via targeting SREBP1. Cell Prolif..

[28] Liu G (2019). Sorafenib kills liver cancer cells by disrupting SCD1-mediated synthesis of monounsaturated fatty acids via the ATP-AMPK-mTOR-SREBP1 signaling pathway. FASEB J..

[29] Chen M (2018). An aberrant SREBP-dependent lipogenic program promotes metastatic prostate cancer. Nat. Genet..

[30] Liu Z (2018). Fractalkine/CX3CR1 contributes to endometriosis-induced neuropathic pain and mechanical hypersensitivity in rats. Front. Cell Neurosci..

[31] Wang Y (2018). Roles of SIRT1/FoxO1/SREBP-1 in the development of progestin resistance in endometrial cancer. Arch. Gynecol. Obstet..

[32] Yamada S, Tsuyoshi H, Tsujikawa T, Okazawa H, Yoshida Y (2019). Predictive value of 16alpha-[18F]-Fluoro-17beta-estradiol PET as a biomarker of progestin therapy resistance in patients with atypical endometrial hyperplasia and low-grade endometrial cancer. Clin. Nucl. Med..

[33] Xu W (2015). Upregulation of mitogen-inducible gene 6 triggers antitumor effect and attenuates progesterone resistance in endometrial carcinoma cells. Cancer Gene Ther..

[34] Zhuo Z, Yu H (2017). miR-205 inhibits cell growth by targeting AKT-mTOR signaling in progesterone-resistant endometrial cancer Ishikawa cells. Oncotarget.

[35] Satyaswaroop PG, Clarke CL, Zaino RJ, Mortel R (1992). Apparent resistance in human endometrial carcinoma during combination treatment with tamoxifen and progestin may result from desensitization following downregulation of tumor progesterone receptor. Cancer Lett..

[36] Chambers JT (1988). Estrogen and progestin receptor levels as prognosticators for survival in endometrial cancer. Gynecol. Oncol..

[37] Sun Y (2015). SREBP1 regulates tumorigenesis and prognosis of pancreatic cancer through targeting lipid metabolism. Tumour Biol..

[38] Lin L (2014). SIRT1 promotes endometrial tumor growth by targeting SREBP1 and lipogenesis. Oncol. Rep..

[39] Perone Y (2019). SREBP1 drives Keratin-80-dependent cytoskeletal changes and invasive behavior in endocrine-resistant ERalpha breast cancer. Nat. Commun..

[40] Wang S (2003). Mechanisms involved in the evolution of progestin resistance in human endometrial hyperplasia–precursor of endometrial cancer. Gynecol. Oncol..

[41] Wang Y (2019). Prolonged conservative treatment in patients with recurrent endometrial cancer after primary fertility-sparing therapy: 15-year experience. Int. J. Clin. Oncol..

[42] Chen X (2009). Aberrant survivin expression in endometrial hyperplasia: another mechanism of progestin resistance. Mod. Pathol..

