# Supplementary material for: Fatostatin reverses progesterone resistance by inhibiting the SREBP1-NF-κB pathway in endometrial carcinoma
Source: Cell Death Dis. 2021 May 26;12(6):544. doi: 10.1038/s41419-021-03762-0 (PMC8155186; doi:10.1038/s41419-021-03762-0)
Supplement: Supplementary file 1 — supplement figure legends [file 41419_2021_3762_MOESM1_ESM.docx]

**Figure Supplementary 1**

**Comparison of resistance of Ish cells and IshMR cells after overexpression of SREBP1**

S1A. Ish-PCMV-SREBP1 cells and IshMR-PCMV-SREBP1 cells were detected viability with different-dose MPA by MTT assay.

S1B. The proliferation capacity of Ish-PCMV-SREBP1 cells and IshMR-PCMV-SREBP1 cells in MPA were demonstrated by EDU assay.

4D. Ish-PCMV-SREBP1 cells and IshMR-PCMV-SREBP1 cells were treated with 0.60μM MPA for 48h respectively. Apoptosis was detected by flow cytometry after staining with FITC Annexin-V and PI.

All experiments were repeated three times at least. **P*< 0.05, ***P*< 0.01, ****P*< 0.001 and *****P*<0.001 for statistical analysis of the indicated groups. Statistical analysis was performed using Student’s t test.

**Table Supplementary 1**

The primers of genes used are shown at Table Supplementary 1.
