# Supplementary figures and images for: Fatostatin reverses progesterone resistance by inhibiting the SREBP1-NF-κB pathway in endometrial carcinoma
Source: Cell Death Dis. 2021 May 26;12(6):544. doi: 10.1038/s41419-021-03762-0 (PMC8155186; doi:10.1038/s41419-021-03762-0)

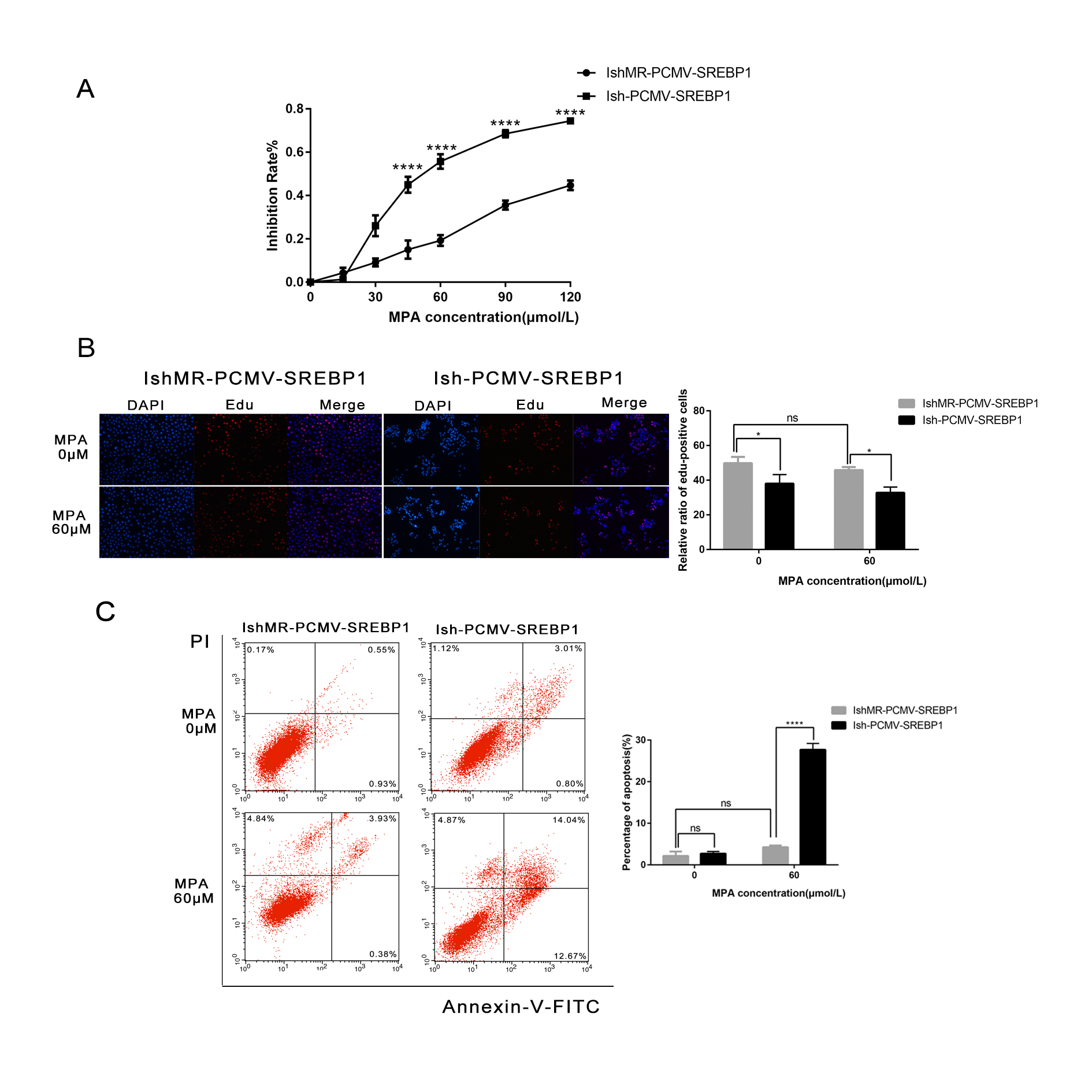

Supplement: Supplementary file 2 — supplement figure 1 [file 41419_2021_3762_MOESM2_ESM.png]
